# Changes in Cardiac Tone Regulation with Fatigue after Supra-Maximal Running Exercise

**DOI:** 10.1100/2012/281265

**Published:** 2011-12-28

**Authors:** Pierre-Marie Leprêtre, Philippe Lopes, Claire Thomas, Christine Hanon

**Affiliations:** ^1^Laboratoire de Recherche Adaptations Physiologiques à l'Exercice et Réadaptations à l'Effort, EA 3300, UFR-STAPS, Université de Picardie Jules Verne, Avenue Paul Claudel, 80025 Amiens cedex 1, France; ^2^Laboratoire de Biomécanique et de Physiologie, Mission Recherche, Institut National du Sport, de l'Expertise et de la Performance(INSEP), 75012, Paris, France; ^3^Département STAPS, UFR Sciences Fondamentales Appliquées, Université d'Evry-Val d'Essonne,91025, Evry, France; ^4^Centre d'étude de la Sensorimotricité (CESeM), UFR Biomédicale, Université Paris Descartes, UMR 8194 CNRS, 45 rue des Saints-Pères, F-75270 Paris Cedex 06, France

## Abstract

To investigate the effects of fatigue and metabolite accumulation on the postexercicse parasympathetic reactivation, 11 long-sprint runners performed on an outdoor track an exhaustive 400 m long sprint event and a 300 m with the same 400 m pacing strategy. Time constant of heart rate recovery (HRR*τ*), time (RMSSD), and frequency (HF, and LF) varying vagal-related heart rate variability indexes were assessed during the 7 min period immediately following exercise. Biochemical parameters (blood lactate, pH, PO_2_, PCO_2_, SaO_2_, and HCO_3_^−^) were measured at 1, 4 and 7 min after exercise. Time to perform 300 m was not significantly different between both running trials. HHR*τ* measured after the 400 m running exercise was longer compared to 300 m running bouts (183.7 ± 11.6 versus 132.1 ± 9.8 s, *P* < 0.01). Absolute power density in the LF and HF bands was also lower after 400 m compared to the 300 m trial (*P* < 0.05). No correlation was found between biochemical and cardiac recovery responses except for the PO_2_ values which were significantly correlated with HF levels measured 4 min after both bouts. Thus, it appears that fatigue rather than metabolic stresses occurring during a supramaximal exercise could explain the delayed postexercise parasympathetic reactivation in longer sprint runs.

## 1. Introduction

Autonomic nervous system function is often assessed in clinical settings by measuring resting heart rate (HR), heart rate variability (HRV), or heart rate recovery (HRR) following exercise [1]. During the last decade, heart rate variability (HRV), that is, a marker of parasympathetic heart rate (HR) modulation, has also been used in exercise physiology to evaluate the fitness level and the physiological responses to physical activity [2–5]. Regulating cardiovascular function to satisfy the metabolic demand of working muscles, the autonomic nervous system has been investigated during recovery after physical exercises performed at different intensities. Kaikkonen et al. [6] and Martinmäki and Rusko [7] observed a delayed postexercise HRV dynamics with increased exercise intensity. Perini et al. [8] already showed a slower recovery of sympathetic activity after an exercise at 70% compared to 21–49% of maximal oxygen uptake value (VO_2max_). Kaikkonen et al. [9] reported a lower HRV after a running event at 93.0% compared to 85.0% of VO_2max_. Parasympathetic reactivation, thus, appears to be mainly related to anaerobic process participation [10].

Metabolic stresses on the exercising skeletal muscle produce cardiovascular change during exercise and its recovery [11–13]. It has been shown that exhaustive supramaximal exercise induced a significant decrease in capillary blood bicarbonate concentration (HCO_3_^−^), pH, base excess (BE), and carbon dioxide pressure (PCO_2_) at exhaustion and during the recovery period [14, 15] which could affect muscle contracting activity and metaboreflex responses compared to aerobic exercise. It has been also reported that high exercise intensity experienced by weightlifters caused muscle's exposure to the products of anaerobic metabolism and a delayed vagal response [16, 17]. However, very few studies looked at the respective anaerobic contribution in the energy production on postexercise parasympathetic reactivation. In our knowledge, only Buchheit et al. [10] examined the effects of lactate production on postexercise parasympathetic reactivation. Although they found a delayed vagal response after a repeated all-out running sprint events (RS) compared to a moderate continuous exercise which inducing a similar mean oxygen uptake, they failed to separate the respective effect of anaerobic contribution and the different levels of fatigue induced by exercise intensity on the parasympathetic response recovery [10].

Although the effect of intensity on parasympathetic reactivation is well documented [6, 7, 10], those of fatigue, related to power decrease during supramaximal exercise, remained unknown. Indeed, the inability to maintain a given level of muscular force at the end of supramaximal exercise would result in a decrease in performance associated with generallocomotor muscle weakness and ultimately system fatigue. For instance, a systematic decrease in speed race can be found during the last 100 m of long sprint events (e.g, 400 m) in top class runners [18–20] that is related to the occurrence of fatigue and, therefore, to the metabolic accumulation during exercise [21]. Therefore, it could be of major interest to determine the effect of metabolic status on the postexercise cardiac tone response independently of exercise intensity in healthy well-trained subjects.

Therefore, to better understand the effect of fatigue on postexercise recovery, and the relative importance of exercise metabolic accumulation on postexercise parasympathetic recuperation, heart rate recovery was compared during nonfatigued and exhaustive supramaximal running bouts in well-trained long sprint runners. We hypothesized that the metabolic and ionic accumulation which occurred during long sprint events rather than exercise intensity could induce a delayed parasympathetic reactivation and a longer heart rate recovery.

## 2. Methods

### 2.1. Subjects

Eleven well-trained long-sprint runners (9 male and 2 women) gave written informed consent to participate in the study, which was conducted in accordance with the guidelines of the declaration of Helsinki. Their anthropometric characteristics included a mean (±SD) age of 21.6 ± 6.3 years, a body weight of 67.0 ± 4.7 kg, a height of 177.7 ± 6.3 cm, and maximal oxygen uptake and heart rate values of 59.8 ± 7.3 mL·min^−1^·kg^−1^ and 194.8 ± 7.9 beats·min^−1^, respectively. The subjects had a mean personal outdoor 400 m race record of 50.9 ± 1.3 s (range from 49 to 60 s).

### 2.2. Experimental Protocol

 During the precompetitive summer season, athletes completed 3 tests on an outdoor track, separated by at least 48 hours and no more than 2 weeks. The tests were performed at the same time of the day, 3 hours after a standardized lunch, with no caffeine taken within the last 48 hours, and identical drink ingestion before both exercises. The environmental conditions were similar for all. The first test consisted of an incremental running exercise (warm-up 10 km·h^−1^ followed by an increment of 1 km·h^−1^ per minute until exhaustion) in order to determine maximal aerobic speed. During the second and the third sessions and after a standardised and supervised warm-up, subjects ran as quickly as possible a 400 m sprint and a 300 m during which the subjects were asked to duplicate the exact pacing strategy of the previous 400 m. To assist the subjects to reproduce the identical pacing regulation of the 400 m running trial, an audio signal generator was programmed to produce individualized pacing for each 25 m during the 300 m exercise [21]. Within 5 s of completing the exercise, subjects sat passively during which time beat-to-beat HR was measured during a 7 min recovery period [2].

### 2.3. Data Recordings

 During each exercise and its subsequent passive recovery phase, heart rate value (HR) was measured beat-to-beat using a Polar S810 heart rate monitor and T61-coded electrode belt (Polar Electro, Kempele, Finland) [22]. 85 *μ*L blood tip was collected from the ear lobe before exercise and at 1, 4, and 7 min after exercise. Blood lactate, pH, PO_2_, PCO_2_, SaO_2_, and HCO_3_ values were measured using the i-STAT portable clinical analyser (Abbott, East Windsor, USA).

### 2.4. Postexercise HR Recovery Assessment

 All R-R series were extracted on an IBM compatible PC using a processing program (Polar Pro Trainer 5.0, Polar Electro, Kempele, Finland). Occasional ectopic beats (irregularity of the heart rhythm involving extra or skipped heartbeats, that is, extrasystole and consecutive compensatory pause) were visually identified and manually replaced with interpolated adjacent R-R interval values. Heart rate recovery (HRR) was calculated by fitting the 7 min postexercise HR recovery data into a first-order exponential decay curve [10]. The time courses of measured HRR were described in terms of exponential functions fitted to the data using nonlinear regression techniques. The calculation of the best-fit parameters was chosen by the program so as to minimize the sum of the squared differences between the fitted function and the observed response (Sigma Plot 10, Jandel, Chicago, IL, USA). Two exponential models were used to describe HRR for each work rate. According to the both following equations, HRR kinetics fit by either mono- (1) or double-exponential (2) function [23]:

(1) $$\begin{array}{cc} \text{HR}\left(t\right) & ={\text{HR}}_{0}-\left[A{}_{1}\;\times \;\left(1-e^{-(t-\text{TD}1)/\text{HRR}\tau 1}\right)\right]\cdot u_{1} \end{array},$$

(2) $$\begin{array}{cc} \text{HR}\left(t\right) & ={\text{HR}}_{0}-\left[A_{1}\;\times \;\left(1-e^{-(t-\text{TD}1)/\text{HRR}\tau 1}\right)\right]\cdot u_{1}\\ & \;-\left[A_{2}\;\times \;\left(1-e^{-(t-\text{TD}2)/\text{HRR}\tau 2}\right)\right]\cdot u_{2}. \end{array}$$

where *u*_1_ = 0 when TD1 = 0 s, *u*_1_ = 1 when TD1 > 0 s, *u*_2_ = 0 when TD2 = 0 s, and *u*_2_ = 1 when TD2 > 0 s. HR(*t*) represents heart rate (beats·min^−1^) at time (*t* in seconds), HR_0_ is the mean HR value at the end of the exercise, *A*_1_ and *A*_2_ are the asymptotic amplitudes for the first and second exponential of heart rate kinetics, HRR*τ*1 and HRR*τ*2 are the time constants, TD1 and TD2 as the time delays of each exponential. 

### 2.5. Time-Varying Vagal-Related HRV Index

While a progressive increase in the R-R interval is generally observed over the initial 5 min of recovery, a piecewise linear curve with superimposed oscillations is common during shorter (i.e., 15–60 s) HR sampling. Thus, a time-varying vagal-related index, the root mean square of successive differences in the R-R intervals (RMSSD) was assessed during 7 min of exercise recovery. To smooth out transient outliers in the HRV plots (HRV versus time in recovery), a median-filter operation was performed in which each value was replaced with the median of the value as well as the preceding and following values. The first and last values were not median filtered [10]. A power frequency analysis (fixed 21 linear sampling; frequency of 2389 equally spaced points per 7 min period) was also performed sequentially with a fast Fourier transform based on a nonparametric algorithm with a Welsh window [9, 24]. The power densities in the LF band (0.04–0.15 Hz) and the HF band (>0.15–0.50 Hz) were calculated from each 7 min spectrum by integrating the spectral power density in the respective frequency bands.

### 2.6. Statistical Analysis

Statistical analyses were carried out using Statview Software 5.0 (SAS Institute Inc., Cary, USA). Descriptive statistics are expressed as mean and standard deviation (SD). According to the data, the normality distribution of the population was analyzed by variance comparison by means of the Fisher Snedecor test. Statistical comparisons over time of the metabolic and HR variables were made using a two-way repeated measures analysis of variance (ANOVA). Paired *t*-tests were used to compare HRR kinetics responses. Scheffe post hoc tests were carried out when appropriate. For bivariate correlations, the Pearson correlation coefficient was calculated. All significant differences were at the *P* < 0.05 level unless stated otherwise.

## 3. Results

The mean time to realize the 400 m running sprint was 52.2 ± 2.4 s and corresponded to 97% of their best performance. The 300 m intermediary time measured during the exhaustive 400 m running trial was not significantly different from the time to perform the 300 m running bout (38.7 ± 1.9 versus 39.2 ± 2.1 s, NS). A significant decrease in running speed (from 2.2 to 15.9%) was found during the last 100 m of 400 m bouts (*P* < 0.05). Expressed as a percentage of the velocity at 300 m, this decrease during the 400 m was 9.9 ± 5.5% (from 2.2 to 20.4%). A running exercise effect was also found on lactate (*P* < 0.01), pH (*P* < 0.01), HCO_3_^−^ (*P* < 0.01), and PCO_2_ (*P* < 0.05) values but not on PO_2_ (NS) and SaO_2_ (NS) (Table 1). Except for SaO_2_ (NS), a recovery time effect was also found on these previous biochemical values (Table 1).

**Figure 1:**
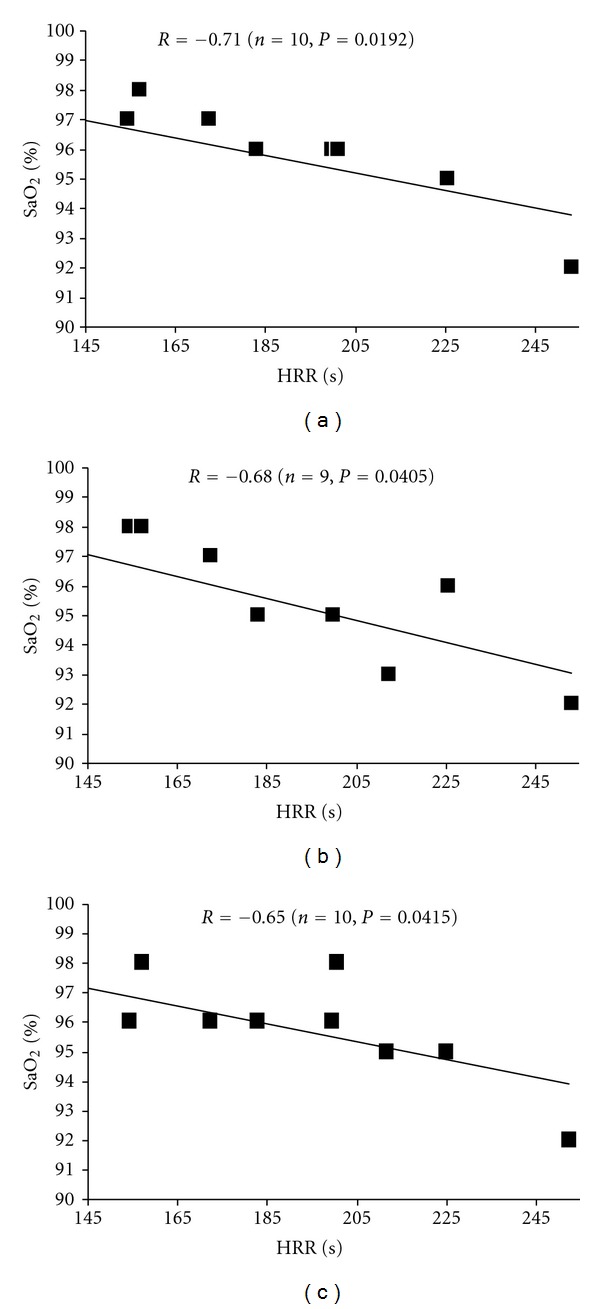
Relationship between heart rate recovery time constant (HRR*τ*) and SaO_2_ values measured at 1 (a), 4 (b), and 7 min (c) recovery after exhausted 400 m running.

**Figure 2:**
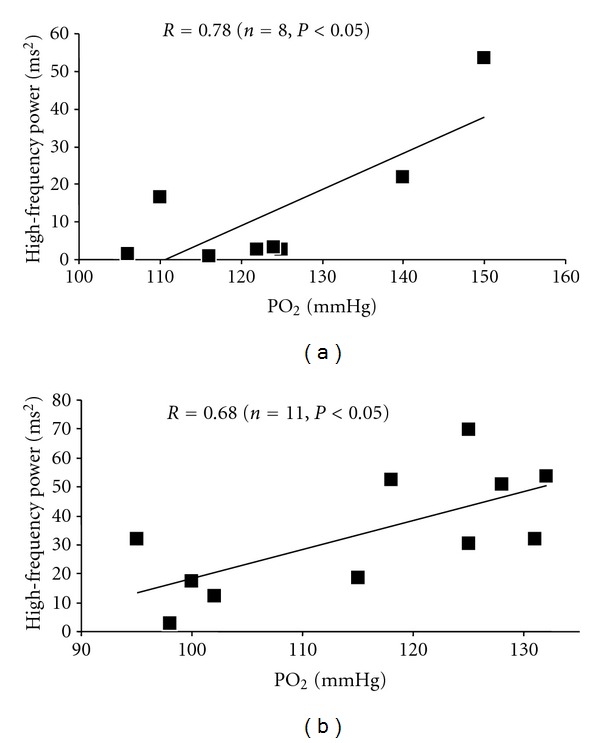
Relationship between partial pressure of oxygen (PO_2_) and high-frequency power (HF) at 4 min 400 m (a) and 300 m (b) postexercise recovery.

During recovery, no significant difference was found in HR amplitude (NS). However, HHR*τ* was longer after the 400 m compared to the 300 running exercise bouts (183.7 ± 11.6 versus 132.1 ± 9.8 s, *P* < 0.01) (Table 1). Absolute power density in the LF and HF bands was lower after 400 m compared to the 300 m running tests (*P* < 0.05 and *P* < 0.05, resp.). Conversely, there was no exercise effect on RMSSD values (NS). LF and RMSSD values were also significantly influenced by the recovery time (*P* < 0.01, Table 1). HRR*τ* values were inversely correlated with HF and LF bands measured at 1 and 4 min after 300 m running exercise (*P* < 0.05) and at 1, 4, and 7 min recovery period after exhausted 400 m bout (*P* < 0.05). Significant relationship was also found between HRR*τ* and SaO_2_ values measured at 1, 4, and 7 min recovery after exhausted 400 m running event (Figure 1). Finally, partial pressure of oxygen (PO_2_) and high frequency bands (HF) were correlated at 4 min 400 m and 300 m postexercise recovery (Figure 2).

## 4. Discussion

The aim of this study was to investigate the effects of fatigue and metabolite accumulation on the postexercice parasympathetic reactivation in well-trained long-sprint runners who performed one exhaustive and another nonfatigued supramaximal running exercises with the same pacing strategy. Significant differences in metabolic values, HRR*τ*, HF, and LF bands were found when comparing both exercise types. Furthermore, our results did not show strong relationship between metabolic responses and postexercise parasympathetic reactivation after both running sprints events with or without fatigue. Firstly, we are going to discuss the occurrence of fatigue during long running sprint events.

In agreement with previous studies [18–20], the subjects of the present study presented a significant decrease in their terminal running speed (ranged from 2.2 to 15.9%) during the last 100 m of the exhaustive 400 m sprint event. Brüggeman et al. [18] also reported a 15.0% decrease in speed race systematically occurring during the last 100 m of long sprint events in top class runners. Thus, this inability to maintain the running speed demonstrating the occurence of fatigue in our subjects. Several studies investigated the effects of system fatigue on running mechanics [25, 26] but the causes of the terminal running speed drop remained unknown. It has been established that exhaustive supramaximal exercise induced a significant decrease in capillary blood bicarbonate concentration (HCO_3_^−^), pH, base excess (BE), and carbon dioxide partial pressure (PCO_2_) at exhaustion and during the recovery period [14]. Recently, a negative correlation was found between running performance, muscular pH, and lactate accumulation rates [15]. In mixed healthy sedentary population performed an exhaustive cycle exercise at 120% of their maximal oxygen uptake, Messonnier et al. [15] reported a greater time to fatigue after oral sodium ingestion compared to control trials. Duffield et al. [27] already showed a significant negative correlation between race performance and anaerobic energy system involvement (lactate/PCr) for the female 400 m events (*r* = −0.87).

To further explore whether muscular fatigue and metabolic accumulation are responsible for the delayed automatic (autonomic???) regulation in trained subjects, it would be ideal to have a simple noninvasive tool that could be used to measure parasympathetic reactivation. In the present study, we used the time constant of heart rate recovery (HHR*τ*) which was measured after the 400 m and the 300 m running exercises. We found longer HRR*τ*  values after 400 m compared to 300 m running bouts. Blood lactate concentration was also higher after 400 m compared to the 300 m events, but no relationship was found between HRR*τ* and lactate values. Recently, Ba et al. [12] observed a negative correlated between heart rate decay and maximal postexercise lactate level or hypoxemia. Darques et al. [28] already showed that supramaximal exercise caused exercise hypoxemia which may induce group IV muscle afferents activation. Furthermore, in eight trained males engaged in 5 km cycling trials, Amann et al. [29] observed that changing arterial oxygen content affected time trial performance and central neural drive, estimated using quadriceps electromyography. In our subjects, although no exercise effect was found on PO_2_ the significant relationship between HRR*τ* and SaO_2_ values during the exhaustive 400 m running recovery raised the question about the implication of O_2_ haemoglobin concentration in response to the central neural drive after exhaustive exercise. Nevertheless, Kannankeril et al. [30] demonstrated that HRR cannot be used as a pure index of parasympathetic reactivation because it is likely mediated by both sympathetic withdrawal and parasympathetic reactivation. Actually, it has been suggested that HR decrease is mediated by sympathovagal balance [23]. Hence, the heart rate variability (HRV) was proposed to evaluate the sympatho-vagal balance during exercise and recovery. For instance, Buchheit and Gindre [4], Goldberger et al. [5] and Buchheit et al. [10] used the root mean square of standard deviations (RMSSD) to investigate the effect of exercise modalities (intensity, continuous versus interval training) on the HRV dynamics. In their blockade study, Goldberger et al. [5] reported a positive correlation between postexercise RMSSD value and parasympathetic activation. They also showed a quick increase in RMSSD after exercise. Also, in the present study, fatigue did not have a significant impact on RMSSD. Thus, parasympathetic reactivation seems to be mainly related to high-intensity exercise. The occurence of the HRV suppression after brief effort was already reported. Buchheit et al. [10] showed a parasympathetic reactivation during the first 10 min period after a moderate isocaloric continuous exercise, whereas parasympathetic nerve remained depressed after two repeated running sprint events. Niewiadomski et al. [31] reported that RMSSD was still weak one hour after a Wingate test compared with submaximal exercise.

Recently, fast Fourier transform (FFT) spectral analysis was used immediately after exercise to evaluate parasympathetic reactivation [6, 32]. In our study, LF and HF were lower after 400 m compared to the 300 m running tests. Considering that vagal activity affects the entire frequency range of HRV, Kaikonnen et al. [6] suggested that the increase in high-frequency power (HF) during the first 5 min of the postexercise recovery may be caused by vagal reactivation after exercise. Martinmäki and Rusko [7] recently proposed that postexercise LF responses reflected the degree of vagal activation or the baroreflex restoration [7, 33]. Mechanisms which may explain HRV postexercise responses still remained uncertain. In the present study, the time to perform the 400 m was longer than 300 m running trial. However, it has been demonstrated that exercise time had no influence on HRV recovery. In fact, Kaikkonen et al. [6] did not find a significant difference in HRV postexercice recovery responses when the running distance doubled (from 3.500 to 7.000 m). However, they reported a lower HRV after a running event at 74% compared to 63% of maximal oxygen uptake value (VO_2max_) [6]. Perini et al. [8] already showed a slower recovery of sympathetic activity after an exercise at 70% compared to 21–49% of VO_2max_. These results were supported by a recent study of Kaikkonen et al. [9] who found a lower HRV after running bouts at 93% compared to 85% of VO_2max_. The chronic low exercise blood flow routinely experienced in the muscle of the 400 m sprinters may cause a chronic exposure to the products of anaerobic metabolism [17] and a delayed vagal response. Thus, parasympathetic reactivation seems to be mainly related to anaerobic process participation during physical exercise. However, neither BE nor lactate response was correlated to LF and HF in our trained subjects. Previously, it has been demonstrated that ATP rather than aerobic or anaerobic processes had a chronotropic effect on cardiac activity. By using magnetic resonance techniques, Pluim et al. [34] showed that heart rate variability is significantly correlated with high-energy phosphate metabolism in trained and sedentary subjects. In fact, intravenous administration of ATP produced a 4-fold increase in the variability of heart rate typically indicating an activation of the parasympathetic myocardial regulation [35]. Therefore, an extra energy cost of skeletal muscle to the detriment of cardiac activity or a disruption of sarcolemmal ATP-sensitive K^+^ channels [36] induced by supramaximal exercise may provoke a delayed cardiac tone response. On the other hand, the aerobic contribution to the energy provisionduring long sprint events may explain the lack of relation between HRV indices and anaerobic metabolic markers. In fact, Duffield et al. [27] reported that the aerobic/anaerobic energy system contribution (AOD method) to the 400 m event was 41/59% and 45/55% in 11 male and 5 female 400 m trained athletes, respectively. However, only PO_2_ and HF values measured 4 min after the 300 and 400 m were correlated in the present study. Fisher and White [11] reported an attenuated muscle afferent feedback in athletic groups participating in training programmes that placed a high anaerobic load on skeletal muscle groups. Thus, it appears that training may decrease the metabolic stimulation of muscle afferents and in some instances, chronic exposure to the products of anaerobic metabolism may blunt the sensitivity of the muscle metaboreflex in well-trained subjects. Hence, the delayed on parasympathetic reactivation after exhaustive supramaximal exercise in trained long-sprint runners still remains unexplained.

## 5. Conclusions

The purpose of the present study was to investigate the effect of fatigue induced by exercise intensity and anaerobic metabolic accumulation on the parasympathetic reactivation. Our results showed that HRV remained lower after an exhaustive compared to nonfatigued running exercise bout, both performed with the same pacing strategies. However, the occurence of metabolic and ionic accumulation did not explain the delayed HRV dynamics after supramaximal exercise in sprint runners. Our results raised the question about the role of fuel efficient on the HRV postexercise response.

## Figures and Tables

**Table 1:** Biochemical and heart rate variability parameters measured during the postexercise recovery.

		1 min recovery		4 min recovery		7 min recovery	
		400 m	300 m	400 m	300 m	400 m	300 m
Biochemical parameters							
Lactate	(Mm)	16.5 ± 1.0	14.6 ± 1.5*	21.2 ± 2.3	16.6 ± 1.3*	22.1 ± 2.0	16.8 ± 1.5
pH		7.1 ± 0.1	7.2 ± 0.0*	7.0 ± 0.1	7.2 ± 0.1*	7.0 ± 0.1	7.2 ± 0.1*
HCO_3_^−^	(Mm)	10.0 ± 1.6	13.5 ± 1.9*	6.5 ± 1.1	10.5 ± 1.7*	5.5 ± 1.4	10.0 ± 2.2*
PO_2_	(mmHg)	114.0 ± 12.3	104.6 ± 17.8	124.1 ± 14.7	115.4 ± 14.2	129.4 ± 20.5	117.6 ± 10.6
PCO_2_	(mmHg)	32.8 ± 3.7	34.8 ± 3.5*	24.5 ± 5.2	29.1 ± 2.6*	23.0 ± 3.7	26.9 ± 4.0*
SaO_2_	(%)	95.9 ± 1.7	96.0 ± 1.7	95.4 ± 2.1	96.9 ± 1.2	95.8 ± 1.7	97.4 ± 0.5

HRV index							
Amplitude	(beats.min^−1^)	74.3 ± 8.0	74.7 ± 10.6				
HRR**τ**	(s)	183.7 ± 11.6	132.1 ± 9.8*				
RMSSD	(ms)	1.8 ± 0.0	1.8 ± 0.1	2.4 ± 0.2	2.1 ± 0.4*	3.3 ± 0.3	3.1 ± 0.1
HF	(ms²)	11.9 ± 21.9	20.6 ± 20.6	15.3 ± 16.1	33.8 ± 20.7*	13.1 ± 12.6	39.7 ± 27.8*
LF	(ms²)	9.3 ± 7.8	49.4 ± 39.3*	24.9 ± 35.9	121.0 ± 117.1*	10.7 ± 6.2	70.8 ± 61.9*

HRR**τ** was the time constant of heart rate recovery and RMSSD, root mean square of the sum of successive differences between adjacent NN intervals. HF and LF represent high- and low-frequency bands, respectively. *Significant difference was less at *P* level <0.05.
